# A deep learning approach for improving spatiotemporal resolution of numerical weather prediction forecasts

**DOI:** 10.1038/s41598-025-17867-5

**Published:** 2025-09-25

**Authors:** Décio Alves, Fábio Mendonça, Sheikh Shanawaz Mostafa, Fernando Morgado-Dias

**Affiliations:** 1 University of Madeira, Campus Universitário da Penteada, Funchal, 9020-105 Portugal; 2 Interactive Technologies Institute (ITI/LARSyS and ARDITI), Edif. Madeira Tecnopolo, Caminho da Penteada piso-2, Funchal, 9020-105 Portugal

**Keywords:** Data fusion, Machine learning, Deep learning, NWP, GFS, ERA5, Atmospheric dynamics, Computer science, Information technology, Climate sciences, Environmental sciences

## Abstract

This study addresses the limitations of traditional numerical weather prediction models in wind forecasting for aviation operations by introducing a deep learning approach based on a spatiotemporal fusion model that enhances the temporal resolution and accuracy of wind forecasts. Specifically, the model integrates Global Forecast System (GFS) with European Centre for Medium-Range Weather Forecasts Reanalysis v5 (ERA5) data, employing a 1-dimensional convolutional layer for spatial data fusion and a bidirectional long short-term memory network for spatiotemporal pattern recognition. The presented approach considerably improves upon the numeric model, increasing temporal resolution from 3-hour to 1-hour intervals and reducing mean absolute error by over 50% for wind speed and direction forecasts. The proposed model achieves 82.85% accuracy in wind direction predictions within a 20° angle, compared to 64.46% for the GFS model forecasts. Case studies demonstrate the proposed model’s superior performance in capturing wind variability, particularly in complex topographical settings like Madeira International Airport. These improvements have relevant implications for aviation safety, flight planning, and fuel consumption optimization. The geographic independence of the proposed approach suggests potential applicability across diverse regions.

## Introduction

Meteorological accuracy, particularly in the domain of wind forecasting, plays an important role in supporting the safety and efficacy of aviation operations^1^. Wind, characterized by its speed and direction, is a dynamic atmospheric element impacting flight schedules, fuel consumption, and overall airport logistics^2,3^. The challenge of predicting wind patterns is emphasized by complex local topographies and rapidly changing atmospheric conditions, which conventional Numerical Weather Prediction (NWP) models, the basis of modern weather forecasting, attempt to manage^4^. Despite their widespread use, these models often encounter difficulties in capturing the rapid variations and localized events typical of wind dynamics, particularly at high temporal and spatial resolutions^4,5^.

The operational constraints of NWP models, due to their significant computational demands, often result in infrequent and delayed forecasts. Typically constrained to one or two operational runs per day, these models depend on extensive computational infrastructure, thus hampering their responsiveness to immediate meteorological changes^4,5^. This limitation is notably critical at locations like Madeira International Airport (LPMA), where unique geographical features contribute to complex wind behaviors that considerably affect flight operations, compounded by strict regulatory wind constraints that can disrupt air traffic^6,7^.

Amid these challenges, a shift towards more responsive and precise forecasting methodologies is needed^8,9^. Recent advancements indicate a new era of enhanced forecasting capabilities that bypass the traditional limitations of NWP models^4,10^. While these improvements are notable across various meteorological applications, they are particularly significant in nowcasting techniques, which aim to provide detailed meteorological insights within a six-hour window^11^. Here, the application of Machine Learning (ML) techniques, traditionally not associated with meteorological sciences, is beginning to redefine the accuracy and efficiency of wind forecasts, particularly those critical for aviation operations^5,12^.

Several studies have highlighted the potential of ML to improve the accuracy of wind speed forecasts of NWP models. Kumar et al. (2019)^13^ and Hoolohan et al. (2018)^14^ have shown that using artificial neural networks and Gaussian process regression can significantly refine predictions of wind speeds, increasing precision by 0.6 m/s over traditional NWP 6-hour forecasts. Supporting this, Zhao et al. (2022)^15^ illustrated that deep learning methods can correct errors in wind speed forecasts derived from NWP, reducing the Mean Absolute Error (MAE) by 0.25 m/s. Additionally, Rozas-Larraondo et al. (2014)^16^ found that nonparametric regression techniques enhance wind speed forecasting at airports, cutting the Root Mean Squared Error (RMSE) from 4.36 m/s to 2.76 m/s at San Sebastian Airport through ML post-processing methods.

Following this work, Han et al. (2022)^17^ developed a new hybrid method for short-term wind speed forecasting that improves on previous models, reducing the MAE by 1.67 m/s, a slight improvement (0.07 m/s) over earlier hybrid models. Du P. (2019)^18^ applied a combined ML technique that merges NWP output with local weather station data, successfully lowering the mean absolute percentage error (MAPE) from 4.57 to 3.74%, at a 3-hour forecast. Wang et al. (2024)^19^ proposed an ML model with spatiotemporal characteristics by using convolutional layers and gated recurrent units, improving the NWP wind speed and direction MAE from 1.63 m/s and 57.93° to 1.37 m/s and 54.82° respectively at 1-hour resolution.

These methodologies have demonstrated potential in correcting the accuracy discrepancies typically found in conventional NWP wind nowcasts, with an emphasis on enhancing both the accuracy of wind speed and direction at fixed gridded values^20^ and improving the spatial resolution at specific points^18,19^.

Recent post-processing studies have concentrated overwhelmingly on spatial super-resolution, for example using U-Net variants to recover kilometre-scale temperature fields from coarse grids^21^. In contrast, systematic attempts to refine the temporal cadence of NWP output remain sparse. Early work by Kumar et al. (2012)^22^ showed that a feed-forward neural network could transform monthly means into six-hourly series while preserving inter-variable correlations above 0.99. More recently, Sinha et al. (2025)^23^ evaluated operator-learning frameworks under zero-shot settings and found that transformer baselines still outperform Fourier Neural Operators when hourly winds are upsampled by factors of 8–15, underscoring the immaturity of methods targeting sub-hour dynamics. Taken together, the literature confirms that temporal downscaling, especially at hour mark resolution or less, remains an underexplored research line.

In this sequence, this study seeks to fill this knowledge gap by exploring NWP spatial-data fusion with Deep Learning (DL) techniques to augment the spatial resolution of wind predictions at airport areas. Particularly, this work aims to increase temporal resolution from 3-hour to 1-hour intervals, allowing to support more precise and timely decision-making in aviation operations^10,24^. Hazards that drive tactical decisions such as convective gust fronts, microbursts, and rapid wind-shear shifts, develop faster than the standard three-hour Global Forecast System (GFS) or even most local scale NWP outputs. Assimilating Mode-S aircraft winds in an hourly rapid-update cycle at Schiphol Airport reduced vector-wind errors in the first 0–3 h of the forecast, confirming the operational value of higher-frequency guidance^25^. At the network scale, convective storms already cause about 7% of Europeanen-route delays, costing airlines roughly €2 billion in 2019^26^.

The machine-learning temporal downscaling pipeline advanced in this study enables wind guidance to be issued on an hourly basis, thereby providing a continuously updated forecast stream and eliminating the six- to twelve-hour latency that characterizes conventional NWP cycles^21,23^. By synchronizing forecast cadence with the rapid evolution of aeronautical hazards and the operational decision horizon of pilots and air traffic controllers, an efficient downscaling model that offers practical guidance for enhancing wind prediction in complex environments that require high-frequency situational awareness is introduced.

This study is divided into four sections. The introduction outlines the scope and objectives of the research. The second section describes the data collection and analytical methods utilized. The results section presents and discusses the main findings, and the final section synthesizes the results and offers concluding remarks.

## Data and methods

### Data sources

This study was based on two distinct databases to access the meteorological data. The first database included forecast data from the National Centers for Environmental Prediction (NCEP) operational GFS. Data were structured on a global latitude-longitude grid with a spatial resolution of 0.25° x 0.25°. Recordings were captured at three-hour intervals, specifically at 3 and 6-hours post-initialization. Model forecasts were generated four times daily at 00, 06, 12, and 18 Coordinated Universal Time (UTC)^27^. The GFS offers fully open global data, delivered four times per day and supported by a continuous archive that begins in January 2015. This combination supplies the volume and timeliness required by our deep learning pipeline and keeps the entire workflow reproducible without licensing barriers. Working with its 0.25 ° grid also magnifies the value added by the spatiotemporal fusion network, while the framework remains model agnostic and can adopt higher-resolution sources as soon as similarly open archives become available.

The second database, the Reanalysis v5 (ERA5) dataset from the European Centre for Medium-Range Weather Forecasts (ECMWF), represents the fifth-generation reanalysis of global climate and weather data. For this analysis, ERA5 used data were re-gridded from their native resolution to a regular latitude-longitude grid at a resolution of 0.25°^28^.

Measurements from both databases included the 10-meter u-component and v-component of wind velocity. The u-component measures the eastward horizontal air velocity at ten meters above the Earth’s surface, expressed in meters per second. The v-component measures the northward horizontal air velocity at the same elevation. These components allow for the calculation of the overall speed and direction of the horizontal wind at 10 m above the surface^29^.

For both databases, the temporal coverage extended from 2015-01-15 03:00:00 UTC to 2024-04-30 21:00:00. This interval represents the maximal period of overlapping data availability from both sources.

For a case study, LPMA wind data was retrieved through Iowa State University’s - Iowa Environmental Mesonet (IEM), which is connected to the National Oceanic and Atmospheric Administration (NOAA) Automated Surface Observing System (ASOS) maintaining a worldwide record of Meteorological Aerodrome Aeronautic Reports (METAR).

ERA5 reanalysis was used as training labels due to its spatial and temporal consistency across the GFS grid points. As a multi‑observationally constrained dataset, ERA5 provides a best‑estimate atmospheric state for the same area targeted by the model. In contrast, LPMA ground observations, being point‑based, were used exclusively for evaluation and case studies, allowing verification of the model’s performance at the actual airport site.

### Data preprocessing

**Fig. 1:**
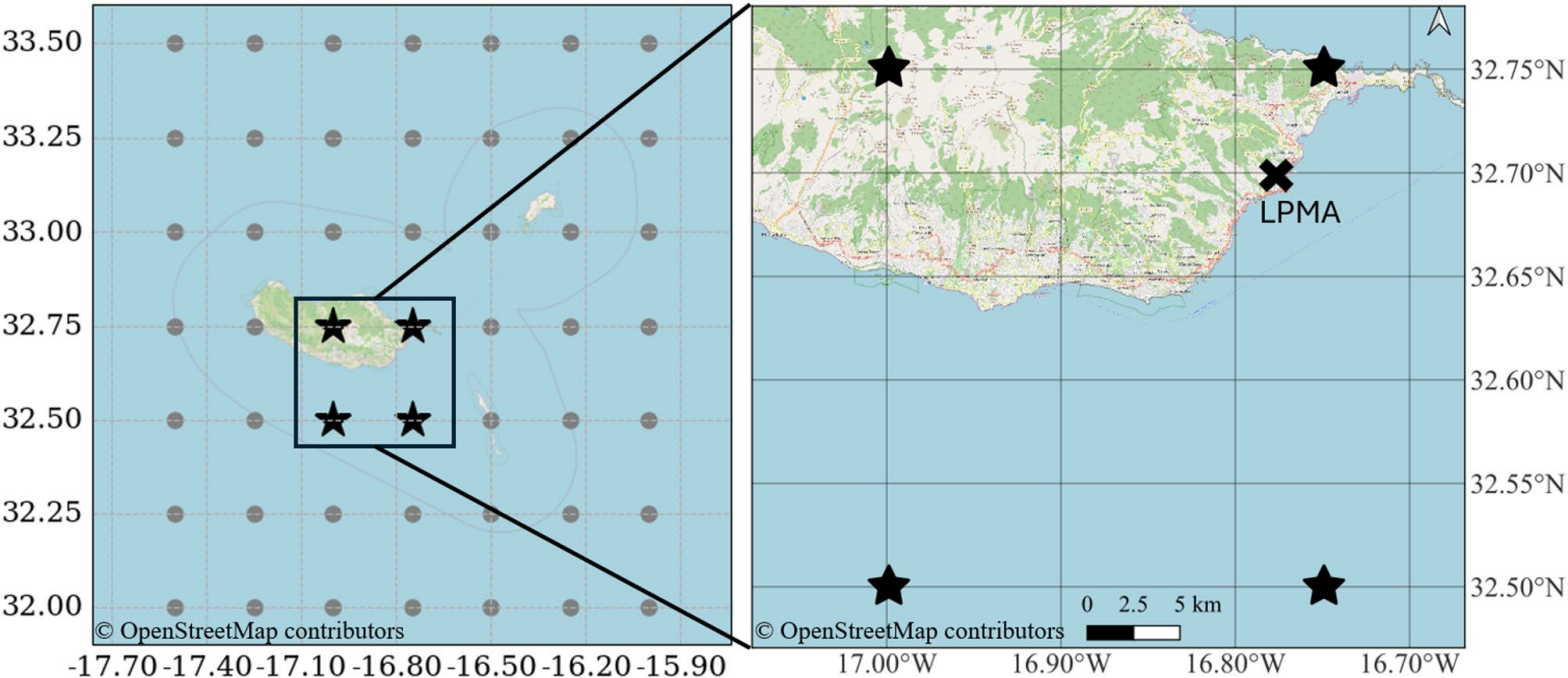
Selected study area, highlighting the grid resolution of the GFS and ERA5 datasets. The figure indicates the four selected grid points with a star symbol and the location of LPMA with a cross symbol. The map was generated by the authors using OpenStreetMap tile data (available at https://www.openstreetmap.org) and Python v3.10.12, available at https://www.python.org).

The initial step involved selecting the four grid points around LPMA from the GFS and ERA5 datasets, which are geographically coincident. These points were chosen to accurately represent local wind conditions and account for the topographical and geographical features unique to the region and are shown in Fig. 1.

The subsequent phase involved a data cleaning process where all non-numeric or faulty values were removed, and a temporal synchronization of the GFS data points with the ERA5 reanalysis values to create a final dataset. At this step, the 3-hour and 6-hour forecasts from each GFS runtime were processed as inputs, and the ERA5 database providing the reanalysis were processed at a 1-hour resolution as labels.

The final dataset covered the period from 2015-01-15 03:00:00 UTC to 2024-04-30 21:00:00. For model training and evaluation, the data were partitioned into three subsets: train data from 2015-01-15 to 2022-01-14, validation data from 2022-01-15 to 2023-01-14, and test data, which was not available to the model at any point of the training and validation steps, from 2023-01-15 to 2024-04-30.

Ingesting two coarse-resolution forecasts from the numerical weather prediction model, separated by three hours (T and T + 3 h), supplies the network with both the instantaneous atmospheric state and its recent temporal tendency. Conditioned on this paired input, the decoder reconstructs intermediate hourly fields at T + 1 h and T + 2 h and simultaneously refines the T + 3 h field, thereby upgrading the guidance from three-hour to hourly without altering the overall lead-time range. The two-snapshot design maintains a compact input tensor compatible with real-time deployment and remains model-agnostic, because any forecast pair that bounds the target window, irrespective of lead time or spacing, can be ingested without architectural modification.

### Data fusion

For model optimization, a comparison was conducted between spatial GFS data fusion techniques and a straightforward input without data fusion. This step involved testing different input time-steps in parallel, ranging from 1 to 10 steps, corresponding to data inputs spanning back from T + 3 to T-27 h.

The data fusion techniques included a 1-dimensional (1D) convolutional layer, which apply convolution operations across the spatial dimension extracting high dimensional features^30^, a time-distributed dense layer approach, which applies dense layers to each time step individually, a generic dense layer involving fully connected neural networks^10^, and an extended Kalman filter (operating as benchmark from conventional methodologies), which are utilized for their ability to recursively estimate the state of a dynamic system by incorporating sequential measurements and accounting for non-linearities^31,32^.

In this context, the fusion integrate input data into a tensor, enhancing predictive capabilities. The 1D convolutional layer extracts high-dimensional features spatially, the time-distributed dense layer applies dense layers to each time step, and the generic dense layer uses fully connected neural networks. An extended Kalman filter fuses sequential measurements to provide refined, accurate estimates by accounting for non-linearities and uncertainties.

The mathematical formulation for the 1D convolutional layer is

1 $$\:y\left(t\right)={\sum\:}_{k=0}^{K-1}x\left(t+k\right)\cdot\:w\left(k\right)+b$$

where $\:y\left(t\right)$ is the output at position $\:t$, $\:x\left(t\right)$ is the input at position $\:t$, $\:w\left(k\right)$ is the weight of the $\:k$-th filter, $\:b$ is the bias term, $\:K$ is the size of the filter^24,30^. The dense layer expression is

2 $$\:y=f\left(W\cdot\:x+b\right)$$

where $\:y$ is the output vector, $\:W$ is the weight matrix, $\:x$ is the input vector, $\:f$ is the activation function and $\:b$ is the bias term. The time-distributed dense layer can be expressed as

3 $$\:{y}_{t}=f\left(W\cdot\:{x}_{t}+b\right)$$

where $\:{y}_{t}$ is the output at time step $\:t$, $\:W$ is the weight matrix, $\:x$ is the input vector, $\:f$ is the activation function and $\:b$ is the bias term.

The extended Kalman filter method can be summarized for prediction as

4 $$\:{\widehat{\text{x}}}_{\text{k}|\text{k}-1}=f\left({\widehat{\text{x}}}_{\text{k}-1|\text{k}-1,}{u}_{\text{k}-1}\right)$$

5 $$\:{P}_{\text{k}|\text{k}-1}={F}_{\text{k}-1}{P}_{\text{k}-1|\text{k}-1}{F}_{\text{k}-1}^{T}+{Q}_{\text{k}-1}$$

where $\:f$ is the state dynamics function, and for the update as

6 $$\:{K}_{k}={{P}_{k|\text{k}-1}{H}_{k}^{T}\left({K}_{k}{P}_{k|\text{k}-1}{H}_{k}^{T}+{R}_{k}\right)}^{-1}$$

7 $$\:{\widehat{\text{x}}}_{\text{k}|\text{k}}={\widehat{\text{x}}}_{\text{k}|\text{k}-1}+{K}_{k}\left({z}_{k}-h\left({\widehat{\text{x}}}_{\text{k}|\text{k}-1}\right)\right)$$

8 $$\:{P}_{k|\text{k}}=\left(I-{K}_{k}{H}_{k}\right){P}_{k|\text{k}-1}$$

where $\:{\widehat{x}}_{k|k-1}$ is the predicted state estimate, $\:{P}_{k|k-1}$ is the predicted covariance estimate, $\:{F}_{k-1}$ is the Jacobian of the state transition function $\:f$, $\:{Q}_{k-1}$ is the process noise covariance, $\:{K}_{k}$ is the Kalman gain, $\:{H}_{k}$ is the Jacobian of the measurement function $\:h$, $\:{R}_{k}$ is the measurement noise covariance, $\:{z}_{k}$ is the measurement at time $\:k$, and $\:I$ is the identity matrix^33^.

The general approach involved applying various data fusion techniques as a preliminary processing step, followed by the base machine learning model, which performed the subsequent state transition. In this way, the data fusion methods served to transform and integrate the input data before it was passed to the core predictive model.

### ML model

A DL model architecture, based on the Time-Series Embeddings from Language Models (TELMo) approach, was employed^10^. This architecture adopts the TELMo approach, employing a stack of bidirectional long short-term memory (BiLSTM) layers followed by timedistributed dense layers. Let $x_{{\text{t}}} \in R^{d}$ be the input at timestep $\:t$. The first BiLSTM layer, with 4d hidden units, computes forward and backward hidden states:$h^{{ - {\text{t}}}} = LSTM^{{\text{f}}} \left( {x_{{\text{t}}} } \right)$ and $h^{{ = {\text{t}}}} = LSTM^{{\text{b}}} \left( {x_{{\text{t}}} } \right)$, yielding the combined state $h_{{\text{t}}} = [h^{{ - {\text{t}}}} ;h^{{ = {\text{t}}}} ] \in R^{{8{\text{d}}}}$.

A timedistributed dense layer applies a pointwise nonlinearity as $z_{{\text{t}}} = \sigma (W_{1} h_{{\text{t}}} + b_{1} )$. This BiLSTM–dense block is repeated twice, yielding two sets of timedistributed features. The outputs of the input and both dense layers are then concatenated as $c_{{\text{t}}} = [x_{{\text{t}}} ;z_{{\text{t}}} ^{{(1)}} ;z_{{\text{t}}} ^{{(2)}} ]$. After flattening across timesteps, a final dense layer $\:y = W_{2} \cdot flatten\left( {c_{{\text{t}}} } \right) + b_{{\text{2}}}$ produces the target prediction, with an output size of 12 units. This design captures multilevel temporal structure akin to Embeddings from Language Models (ELMo) embeddings in natural language, providing a rich, contextaware representation of the input sequence^10^.

**Fig. 2:**
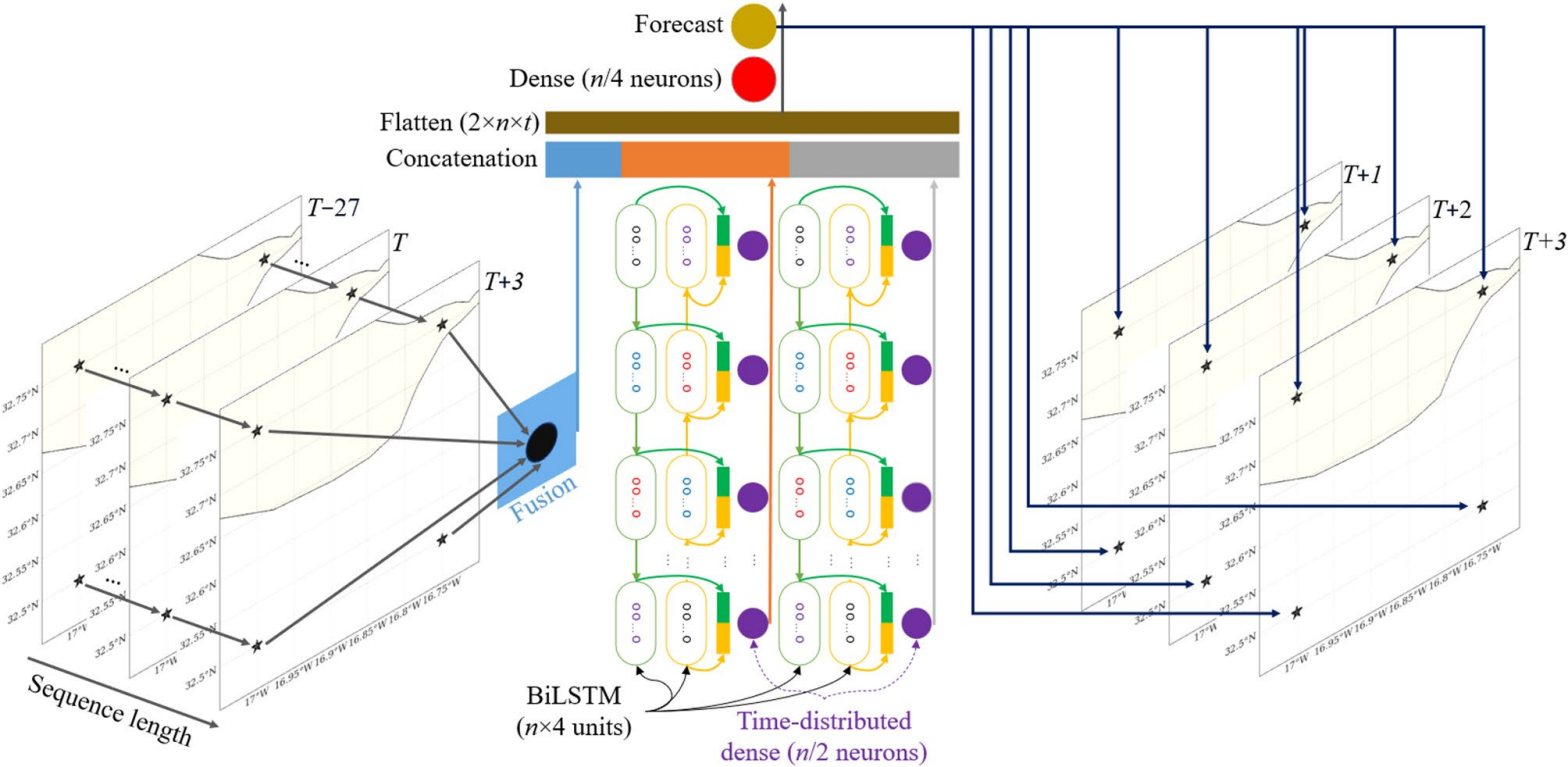
Proposed Spatiotemporal Fusion TELMo (STF-T) architecture.

As illustrated in Fig. 2, the input comprises both temporal and spatial dimensions, encompassing multiple timesteps across four gridded locations. The model then produces a direct three‑step temporal forecast for each of these four spatial points, yielding a high‑resolution, multi‑site prediction of wind conditions.

The model was implemented using the TensorFlow Keras framework and trained with the Adam optimization algorithm, employing a batch size of 512 and a maximum of 100 epochs. The optimization objective was the Mean Squared Error (MSE) loss, with an early stopping mechanism configured to halt training if the validation loss failed to improve over ten consecutive epochs, thereby restoring the best-performing weights. The input sequence comprised two temporal steps, representing a 6‑hour window, yielding a target output of 12 variables (three hourly forecasts across four spatial points). This configuration was selected to balance temporal depth and spatial granularity, while mitigating overfitting and optimizing generalization performance across the targeted prediction horizon.

### Performance analysis

To calculate the performance of the final model, the u-component and v-component of the wind were converted to wind speed in meters per second and wind direction in degrees, using:

9 $$\:\text{wind speed}=\sqrt{{u}^{2}+{v}^{2}}$$

10 $$\:\text{wind direction}={\text{tan}}^{-1}\left(\frac{v}{u}\right)$$

The performance metrics used on this work were the MAE (m/s), MSE (m^2^/s^2^) and RMSE (m/s), following

11 $$\:{\text{MAE}} = \left\{ {\begin{array}{*{20}c} {\frac{1}{m}\sum {_{{i = 1}}^{m} } \left| {Y_{i} - \hat{Y}_{i} } \right|,\:if\:Y_{i} ,\hat{Y}_{i} \:in\:m{\text{/}}s} \\ {\:\frac{1}{m}\sum {} _{{i = 1}}^{m} \left| {\left( {Y_{i} - \hat{Y}_{i} } \right) - 360 \times \left\lfloor {\frac{{Y_{i} - \hat{Y}_{i} + 180}}{{360}}} \right\rfloor } \right|,if\:Y_{i} ,\hat{Y}_{i} \:{\text{in}}\:{\text{degrees}}} \\ \end{array} } \right.$$

12 $$\:{\text{MSE}}\: = \:\left\{ {\begin{array}{*{20}c} {\frac{1}{m}\sum {\:_{{i = 1}}^{m} } \left( {Y_{i} - \hat{Y}_{i} } \right)^{2} ,\:if\:Y_{i} ,\hat{Y}_{i} \:{\text{in}}\:{\text{m/s}}} \\ {\:\frac{1}{m}\sum \: _{{i = 1}}^{m} \left( {\left( {Y_{i} - \hat{Y}_{i} } \right) - 360 \times \left\lfloor {\frac{{Y_{i} - \hat{Y}_{i} + 180}}{{360}}} \right\rfloor } \right)^{2} ,if\:Y_{i} ,\hat{Y}_{i} \:in\:degrees} \\ \end{array} } \right.$$

13 $$\:{\text{RMSE}}\: = \:\left\{ {\begin{array}{*{20}c} {\frac{1}{m}\sum {\:_{{i = 1}}^{m} } \sqrt {\left( {Y_{i} - \hat{Y}_{i} } \right)^{2} } ,\:if\:Y_{i} ,\hat{Y}_{i} \:in\:m/s} \\ {\:\frac{1}{m}\sum \: _{{i = 1}}^{m} \sqrt {\left( {\left( {Y_{i} - \hat{Y}_{i} } \right) - 360 \times \left\lfloor {\frac{{Y_{i} - \hat{Y}_{i} + 180}}{{360}}} \right\rfloor } \right)^{2} } ,if\:Y_{i} ,\hat{Y}_{i} \:in\:degrees} \\ \end{array} } \right.$$

were $\:{Y}_{i}$ represents the reanalysis values, which are the ERA5 1-hour resolution gridded data, $\:{\widehat{Y}}_{i}$ represents the predicted values, and $\:m$ is the number of observations^10,34^.

### Code availability

Developed code is available at DOI 10.5281/zenodo.16903952 with no access restrictions.

## Results and discussion

### Data fusion and input sequence

**Fig. 3:**
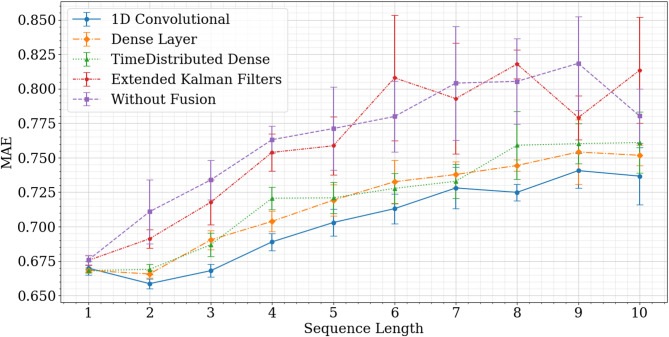
Sequence length MAE comparison between GFS spatial data fusion techniques.

The MAE results from the input length optimization and data fusion methods are shown in Fig. 3. The results suggest that for the spatial input window of 4 points, which is the same size as the spatial output window, the absence of data fusion methods indicates that the model fails to benefit from temporal analysis, with the best result occurring at a sequence length of 1. The extended Kalman filter method, although exhibiting improved performance, still demonstrates that the model does not gain from increasing past information, as the optimal value is achieved with an input sequence length of 1.

While the performance of both the dense layer and the time-distributed dense layer shows slight improvement when utilizing two sequences of data as input, the 1D convolutional layer exhibits a considerably higher increase in performance (and a small improvement when using three sequences). It was, therefore, possible to achieve the lowest MAE with a 6-hour input, corresponding to two sequences of spatial data demonstrating the capability of incorporating spatiotemporal information for GFS spatial data fusion.

### Performance analysis

According to the previous findings, the final model uses the 1D convolutional as GFS spatial data fusion where it processes the input of 2 steps of the 3-hour GFS forecasts and, passing through the DL model, outputs a 1-hour temporal resolution forecast.

In Table 1, the error metrics from the best iteration of the proposed methodology are presented, comparing the GFS 3-hour resolution metrics with the STF-T model 1-hour resolution forecasts.

**Table 1:** Error metrics of the STF-T and GFS models for each Spatial point.

Point	Metric	u-Component		v-Component		Wind speed		Wind direction	
		GFS	STF-T	GFS	STF-T	GFS	STF-T	GFS	STF-T
32.50 *N*−16.75 E	MAE	0.92	0.44	0.87	0.48	1.16	0.46	24.15	13.28
	MSE	1.27	0.34	1.08	0.38	1.77	0.36	1542.04	573.44
	RMSE	1.13	0.58	1.04	0.62	1.33	0.6	39.27	23.95
32.75 *N*−16.75 E	MAE	1.57	0.69	1.46	0.75	1.47	0.70	36.30	17.53
	MSE	3.93	0.83	3.90	0.90	3.42	0.81	3315.76	921.92
	RMSE	1.98	0.91	1.98	0.95	1.85	0.90	57.58	30.36
32.50 *N*−17.00 E	MAE	1.29	0.68	1.43	0.68	1.63	0.66	17.59	13.28
	MSE	2.79	0.79	3.11	0.79	3.93	0.73	1126.92	673.25
	RMSE	1.67	0.89	1.76	0.89	1.98	0.86	33.57	25.95
32.75 *N*−17.00 E	MAE	1.45	0.59	0.88	0.63	0.96	0.60	25.90	13.78
	MSE	3.00	0.58	1.43	0.67	1.54	0.59	1395.58	661.88
	RMSE	1.73	0.76	1.20	0.82	1.24	0.77	37.36	25.73

The comparison of metrics between the GFS and STF-T models for different coordinates in Table 1 reveals several prominent trends. Across all points, the STF-T model consistently demonstrates lower values in MAE, MSE, and RMSE for the u-component, v-component, wind speed, and wind direction metrics compared to the GFS model. Specifically, the STF-T model shows substantial reductions in the error measurements, with MAE values often less than half of those reported by the GFS model. For instance, at 32.75°N, -17.00°E, the proposed model records an MAE of 0.59 m/s for the u-component and 0.6 m/s for wind speed, in contrast to the GFS model values of 1.45 m/s and 0.96 m/s, respectively. Additionally, MSE and RMSE values reflect a similar trend, where the STF-T model errors are distinctly lower, indicating enhanced prediction accuracy. This pattern is particularly evident in wind direction metrics, where the STF-T model shows a noteworthy reduction in error, with RMSE values of 25.73° at 32.75°N, -17.00°E, compared to the GFS 37.36°.

**Fig. 4:**
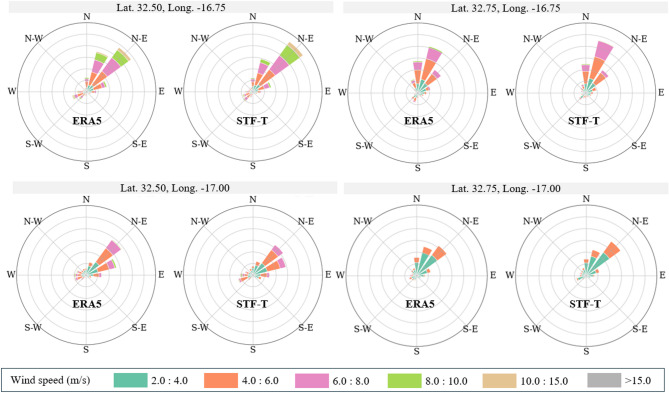
Wind roses of the ERA5 reanalysis and STF-T forecasts for each spatial point.

In Fig. 4, the wind roses depicting the reanalysis ERA5 and the proposed model forecasts for the entire test dataset are depicted, where it can be observed that the STF-T was able to capture and predict the dynamics for each point independently and accurately.

Table 2 provides an overview of the proposed model and the GFS metrics for the spatial mean values of the entire test dataset.

**Table 2:** Error metrics for STF-T and GFS models.

Variable	MAE	MSE	RMSE
u-Component			
GFS	1.31	2.75	1.66
STF-T	0.60	0.63	0.80
v-Component			
GFS	1.16	2.38	1.54
STF-T	0.64	0.69	0.83
Wind speed			
GFS	1.30	2.66	1.63
STF-T	0.61	0.62	0.79
Wind direction			
GFS	25.99	1845.07	42.95
STF-T	14.47	707.62	26.60

The results in Table 2 emphasize the capability of the STF-T model to improve the GFS model forecasts across all variables. The STF-T model consistently shows expressively lower errors, with the MAE values achieving an improvement superior to 100% from the GFS model, indicating greater prediction accuracy. The STF-T model also has a wind direction accuracy within a 20º angle, as defined by the International Civil Aviation Organization^35^ of 82.85%, compared to 64.46% for the GFS model. The proposed model also provides a finer time resolution of 1-hour, compared to the base GFS model 3-hour resolution, offering more timely data updates. These results suggest that the STF-T model offers a significant improvement over GFS forecasts, providing not only a threefold increase in time resolution from 3-hour to 1-hour but also reducing errors by more than half in several metrics.

### Case studies

**Fig. 5:**
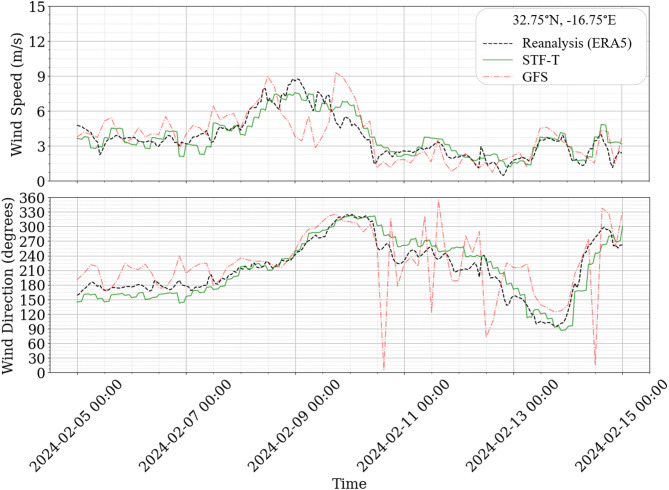
GFS and STF-T wind speed and direction forecasts compared with ERA5 reanalysis data from 05-02-2024 at 00:00 UTC to 15-02-2024 00:00 UTC.

To conduct a more focused temporal analysis of the model performance, a 10-day case study was undertaken at the grid point nearest to the airport (32.75°N, − 16.75°E). This period was selected due to its significant wind speed variability, with reanalysis values ranging from 0.48 m/s to 8.77 m/s, accompanied by wind direction fluctuations spanning 241°. Figure 5 illustrates the results, presenting a comparative analysis of wind speed and direction forecasts generated by the GFS and the STF-T model, compared to the ERA5 reanalysis.

Figure 5 clarifies that the forecasts generated by the proposed model exhibit enhanced alignment with the reanalysis data, while also achieving higher resolution. Specifically, at 2024-02-09 00:00 UTC, the forecasts from the GFS model diverge markedly from the observed reanalysis, whereas the STF-T model demonstrates a superior capability to adjust these forecasts to more closely match the actual measurements.

During the analyzed timeframe, the GFS model recorded MAE, MSE, and RMSE for wind speed of 1.14 m/s, 2.50 m²/s², and 1.58 m/s, respectively. In contrast, the STF-T model achieved significantly lower values of 0.67 m/s, 0.69 m²/s², and 0.83 m/s for the same metrics.

Regarding wind direction, the STF-T model forecasts also aligned more closely with the reanalysis, particularly from 2024-02-10, onward, where the GFS model forecasts began to deviate. For this variable, the GFS model exhibited MAE, MSE, and RMSE values of 34.46º, 2248.81º², and 47.42º, respectively. The STF-T model, however, demonstrated improved performance with corresponding values of 18.09º, 536.67º, and 23.17º.

**Fig. 6:**
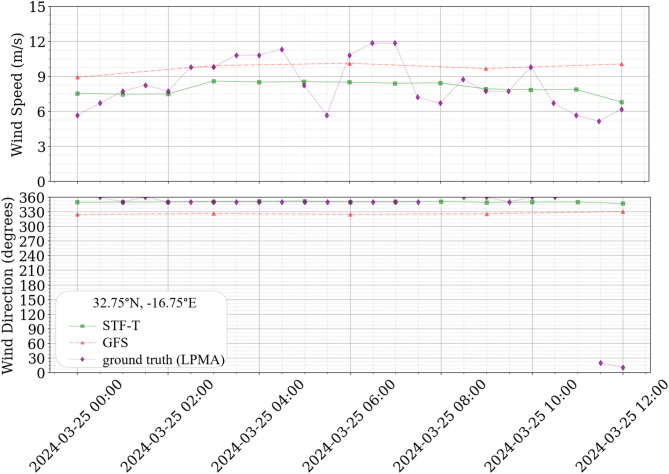
Comparative analysis of ground truth and forecast models from 00:00 UTC to 12:00 UTC on 2024-03-25.

Figure 6 presents a detailed 12-hour comparative analysis spanning from 00:00 UTC to 12:00 UTC on 2024-03-25. This analysis directly compares data from the same grid point to observations recorded at the LPMA wind station, which serves as the ground truth. During this period, the wind station recorded a variation in wind speed from 6.17 m/s to 11.83 m/s. There was a directional shift in the wind from 350° to 2°, amounting to a total angular variation of 3°.

In this more detailed period, it is possible to observe that enhancements in the GFS resolution of 5 temporal forecasts, which the STF-T model successfully augmented to 13, also improved the correlation between the analyzed grid point and the LPMA airport data.

The graphical analysis, illustrated in Fig. 6, demonstrates that the STF-T model aligns more closely with the LPMA observed data. The metrics computed for the STF-T model were MAE of 1.28 m/s, MSE of 2.83 m²/s², and RMSE of 1.68 m/s for wind speed, and MAE of 4.89°, MSE of 76.48°², and RMSE of 8.75° for wind direction. In comparison, the GFS model underperformed, with corresponding metrics of 1.66 m/s MAE, 4.80 m²/s² MSE, and 2.19 m/s RMSE for wind speed and 30.87° MAE, 994.05°² MSE, and 31.53° RMSE for wind direction.

During the specified timeframe, the directional accuracy of the STF-T model reached 90%, indicating a high level of alignment with observed data. In contrast, none of the GFS model forecasts fell within a 20-degree margin of the observed values, resulting in a directional accuracy of 0%.

In interpreting these results, it is important to recognize that the airport observations have a higher temporal granularity (30‑minute records) than either of the model outputs, which operate at hourly (STF‑T) and three‑hourly (GFS) timesteps. As a result, very rapid intra‑hour fluctuations present in the observations are not fully resolved by any of the modeling approaches. Nevertheless, the STF‑T method provides a closer approximation of the observed wind behavior across the available hourly windows, offering a marked improvement over the parent GFS despite this inherent limitation.

## Conclusion

This study introduced a novel approach to enhancing wind forecasting for aviation operations, addressing the limitations of traditional NWP models through the application of advanced machine learning techniques. The proposed STF-T DL model demonstrated significant improvements in both the temporal resolution and accuracy of wind forecasts compared to the base GFS model forecasts.

The key findings of this research include the successful implementation of the STF-T model, which increased the temporal resolution of forecasts from 3 h to 1 h, thus providing more frequent and timely updates crucial for aviation operations. Substantial improvements in forecast accuracy were observed across all metrics, with the STF-T model reducing the MAE by over 50% for wind speed and direction forecasts compared to the GFS model. Furthermore, the accuracy of wind direction within a 20° angle, as defined by International Civil Aviation Organization (ICAO) standards, improved from 64.46% with the GFS model to 82.85% with the STF-T model. Furthermore, case studies demonstrated the STF-T model’s superior performance in capturing wind variability and aligning more closely with ground truth data, particularly during periods of significant wind fluctuations.

These results have important implications for aviation safety and efficiency. The enhanced temporal resolution and improved accuracy of wind forecasts can significantly aid in flight planning, fuel consumption optimization, and overall airport logistics. The STF-T model’s ability to better capture local wind dynamics is particularly valuable for airports in complex topographical settings, such as LPMA location in the south-east coast of Madeira Island.

Certain limitations of this study arise from its reliance on GFS model data as the foundational input for forecast generation, potentially propagating inherent inaccuracies associated with the NWP model. While this investigation did not assess these aspects, the model employs spatial grid points to enhance GFS forecasts. As GFS data are available globally, this approach can be readily transferred to other geographic locations, as demonstrated in prior work by Alves et al.^36^, highlighting its broad applicability across diverse regions.

Future research could explore the integration of real‑time local weather station data providing higher spatial resolution to the forecasts and the use of data augmentation techniques to better generalize the model capabilities, as well as extending the spatial data fusion to include a larger number of points to capture a wider range of meteorological influences while assessing the associated computational requirements and feasibility for operational deployment.

## Data Availability

The datasets used in this study are publicly available from open-access repositories. The Global Forecast System (GFS) operational forecast data were obtained from the National Centers for Environmental Prediction (NCEP) and are available at the Research Data Archive of the National Center for Atmospheric Research: https://doi.org/10.5065/D65D8PWK. The ERA5 reanalysis dataset was retrieved from the Copernicus Climate Change Service (C3S) Climate Data Store: https://doi.org/10.24381/cds.adbb2d47. For the case study, surface wind observations from Madeira International Airport (LPMA) were accessed via the Iowa Environmental Mesonet (IEM), which provides data from the NOAA Automated Surface Observing System (ASOS): https://mesonet.agron.iastate.edu/request/download.phtml. All processed datasets supporting the findings of this study are available from the corresponding author upon reasonable request.
